# "The Greatest Sin against Our Country"

**Published:** 1916-02-05

**Authors:** 


### "The Greatest Sin against our Country."

At the annual general meeting of the London County and Westminster Bank, held on January 27, Mr. Walter Leaf, the deputy-chairman, who presided, stated that no fewer than 1,000 of their staff had been released and were on active service, and 1,100 more had been attested, many of whom had already joined the Colours. To their deep regret they had to record that they had lost no less than thirty killed and eighty-two wounded. Turning to the financial side of the work during the past year, Mr. Leaf called attention to the manner in which the financial machine had adjusted itself to the new conditions and the smoothness with which it had worked.

The bank had the confidence of their customers, the foundation on which all banks must rest. He hoped that the bank in return had done its duty by them, and assisted them in full measure to all their legitimate needs. Their aggregate deposits were nearly eight millions in excess of those which the bank showed a year ago—a far larger sum than had ever appeared on any of their annual statements. He urged upon all the desirability of investing now in the Five per Cent. Exchequer Bonds.

The bank had paid a dividend of 18 per cent. without touching their reserve, which still stood at £4,000,000. Mr. Leaf concluded his speech by an exhortation to all classes to cut down every luxury, and not only to remember but to preach abroad that waste was now the greatest sin against our country, that the war had become a war of saving against waste, and that everyone was a soldier in that war.

